# Screening for postpartum depression at well child visits: evaluating the impact of Michigan’s statewide initiative

**DOI:** 10.1186/s13561-025-00671-2

**Published:** 2025-08-26

**Authors:** Janet Currie, Anna Malinovskaya

**Affiliations:** Department of Economics, Yale University, 30 Hillhouse Ave., New Haven, CT 06511 USA

**Keywords:** Post-partum depression, Screening, Well-child visit, Medicaid

## Abstract

**Objective:**

To examine a 2018 rule change allowing pediatric providers to bill the child’s Medicaid ID for post-partum depression (PPD) screening of mothers conducted during well-child visits, and document its relationship with PPD treatment and infant hospitalizations.

**Study setting and design:**

Screening rates during well-child visits are calculated at the zip code level and used in linear probability and Instrumental Variable (IV) models to examine increases in screening after the policy change and relate them to PPD treatment and infant hospitalizations.

**Data sources and analytic sample:**

Individual-level Medicaid claims were used to compute PPD screening rates and measures of PPD treatment and infant hospitalization.

**Principal findings:**

The policy was associated with increases in screening rates, although take up was uneven and overall screening rates remained low at 8.8%. There was little overall increase in treatment, although in zip codes in the top third of screening rates, higher screening was associated with 10.1% higher probability of maternal treatment. Zip codes with high fractions in poverty and/or minority had low screening rates, but screening was more likely to be associated with increases in treatment in these areas. There are no effects in the full sample of children, but among children above the poverty line, the observed increases in screening reduced the probability of infant hospitalization in the first six months by 7.7%.

**Conclusions:**

The policy change had only limited success increasing screening, but increased screening could lead to more maternal PPD treatment and lower infant hospitalization rates if accompanied by expanded access to PPD treatment.

**Supplementary Information:**

The online version contains supplementary material available at 10.1186/s13561-025-00671-2.

## Introduction

Maternal postpartum depression (PPD) is the leading cause of maternal morbidity with an estimated 1 in 8 postpartum women in the U.S. reporting symptoms in 2018 [1]. Rates of PPD are higher among non-Hispanic American Indian/Alaska Native women (22%), non-Hispanic Asian/Pacific Islander women (19.2%), and non-Hispanic Black women (18.2%), compared to 11.4% among non-Hispanic White women [1]. Non-white women also experience stark disparities in receiving a diagnosis and treatment [2, 3]. PPD increases women’s risk of self-harming behaviors [4, 5] and negatively impacts infant development [6–9]. Yet, despite the importance of PPD, screening rates have remained stubbornly low, with less than half of post-partum women ever being screened [10].

New mothers typically visit a pediatrician multiple times during an infant’s first year. Hence, screening mothers during children’s visits could be an effective way to detect PPD. Given negative impact of PPD on infants and mothers, the American Academy of Pediatrics (AAP) recommends that mothers be screened for PPD at the infant’s first, second, fourth, and sixth-month well child visits [11].

In December 2017, Michigan’s Department of Health and Human Services (MDHHS) released new guidelines, effective January 1, 2018, about PPD screening. After re-iterating that providers should screen for maternal depression during well child visits as recommended by the AAP, MDHHS established procedures allowing providers to bill for such screenings using the infants’ Medicaid identifier (ID) instead of the mothers’ Medicaid ID, and specified that providers use an existing billing code (CPT code 96161) for such screenings [12]. This billing code 96161 had been introduced earlier in 2017 to allow providers to bill for *caregiver* health risk assessments that were deemed to benefit the child; there was no analogous billing code prior to 2017 [13, 14]. Thus, the policy did not establish new guidelines for PPD screening, but codified a way for providers to bill for this service. This policy had the potential to increase postpartum depression screening because mothers are more likely to attend their child’s well child visits than their own postpartum check-up visits [15]. It is less clear, however, whether the policy was likely to increase *treatment* for postpartum depression. At this time, Medicaid-eligible postpartum women were covered for 60 days after giving birth, and women who lost Medicaid coverage after this period might have had difficulty financing treatment. Postpartum Medicaid coverage has since been extended to 12 months.^1^

This study examines the relationship between this billing policy change and maternal PPD screening rates, maternal depression treatment, and child health outcomes. The main child outcome examined is infant hospitalization, which has previously been linked to PPD [17–19].^2^ We use the universe of Medicaid claims data for Michigan postpartum women with deliveries in 2017 and 2018 and all claims for infants born in 2017–2018. The infant sample is larger than the mother sample because Medicaid coverage of infants is more generous in Michigan.^3^ The large sample allows us to examine subsets of the data to see how treatment rates vary across areas that differ in terms of poverty levels and percent minority, as well as whether infant health outcomes vary with the poverty status of the mother.

We find that the release of the guidelines by MDHHS was followed by a doubling of the rate of caregiver screenings during well child visits, though the overall sample-weighted screening rate was still only 8.8% for all mothers, varying from 0% to over 70% across zip codes. Areas that experienced significant increases in screening also saw increases in postpartum depression treatment. Zip codes in the top tercile of screening in 2018 (mean screening rate of 20.1%) saw increases in PPD treatment of 0.088 percentage points, a 10.1% increase over the overall baseline treatment rate in those areas of 17.5%. The relationship between screening and treatment is larger in high minority and high poverty zip codes, even though those areas experienced smaller increases in screening rates after the policy change. Finally, using area-level screening rates as an instrument for whether individual caregivers were screened, we show that when caregivers are screened during their children’s well child visits, the children are less likely to be hospitalized in the first six months of life, but only if their mothers are likely to have had access to treatment.

This study contributes to a relatively small literature on postpartum depression screening during well child visits. Earlier studies have asked whether screening during well child visits was likely to detect cases of PPD that would not have otherwise been detected [20, 21], and whether PPD screening can be integrated successfully into a pediatric practice [22, 23], but there is less evidence about the relationship between screening and health outcomes. We follow the Strengthening the Reporting of Observational Studies in Epidemiology (STROBE) guidelines in this study.

## Empirical strategy

We first document variations in screening rates across zip codes, which are the smallest geographical unit in our data. Focusing on zip codes allows us to control for possible determinants of variations in treatment such as local poverty rates and the percent minority. Because mothers and children cannot be linked in our data, we do not know whether an individual mother’s screening was billed under her child’s ID. Hence, for mothers, we use linear probability models to ask whether their PPD treatment rates are related to zip code PPD screening rates.

For children, we *do* observe whether an individual child’s mother had a screening for PPD that was billed to the child’s Medicaid ID during a well-child visit. Hence, for the child hospitalization outcome, we estimate Instrumental Variables (IV) models using the zip-code-level PPD screening rates as an instrument for the mother having been screened. The maintained assumptions involved in the instrumental variables estimation are that an individual mother is more likely to be screened in an area with high screening rates, and that higher area-level screening rates affect infant health outcomes only by increasing the probability that an individual mother is screened. The inclusion of zip code fixed effects makes it more likely that this exclusion restriction is met, by controlling for all characteristics of the local area that were fixed between 2017 and 2018.

After showing that the rule change was followed by an increase in screening rates, the next step is to examine the association between increases in screening rates at the local zip code level and the probability that an individual woman received treatment for depression (medication and/or psychotherapy) in the six months following the birth. This question is explored using linear probability models of the following form:

1 $$\eqalign{{\rm{Treatmen}}{{\rm{t}}_{{\rm{it}}}}\>{\rm{ = }} & {\beta _{\rm{0}}}\>{\rm{ + }}\>{\beta _{\rm{1}}}{\rm{ScreeningRat}}{{\rm{e}}_{{\rm{zt}}}}\>{\rm{ + }}\>{\beta _{\rm{2}}}{\rm{DepHis}}{{\rm{t}}_{\rm{i}}}\>{\rm{ + }} \cr & {\beta _{\rm{3}}}{\rm{OMental}}{{\rm{H}}_{\rm{i}}}\>{\rm{ + }}\>{\beta _{\rm{4}}}{\rm{FullYea}}{{\rm{r}}_{\rm{i}}}\>{\rm{ + }} \cr & {\beta _{\rm{5}}}{\rm{Hispani}}{{\rm{c}}_{\rm{i}}}\>{\rm{ + }}\>{\beta _{\rm{6}}}{\rm{Povert}}{{\rm{y}}_{\rm{i}}}\>{\rm{ + }}\>{\beta _{\rm{7}}}{\rm{AgeFE}}\>{\rm{ + }} \cr & {\beta _{\rm{8}}}{\rm{ZipFE}}\>{\rm{ + }}\>{\beta _{\rm{9}}}{\rm{2018}}\>{\rm{ + }}\>{\varepsilon _{{\rm{it}}}}, \cr} $$

where *Treatment*_*it*_ is an indicator equal to one if mother *i* received treatment in year *t* and zero otherwise; *ScreeningRate*_*zt*_ is the zip code level screening rate in zip code *z* and year *t* (please see the Data section for details); *β*_*2*_*DepHist*_*i*_ is an indicator equal to one if mother *i* had previously received treatment for depression (which would place her at greater risk of PPD); *OMentalH*_*i*_ is a similar indicator for a history of other mental health conditions (specifically, a history of new filled prescriptions for antipsychotics, antianxiety medications, sedatives, or stimulants); *FullYear*_*i*_ is an indicator equal to one if the mother was covered by Medicaid for the full year, i.e. outside the pregnancy interval, which might indicate that she had access to mental health screenings using her own Medicaid ID as well as mental health treatment; *Hispanic*_*i*_ is a fixed effect for reporting Hispanic ethnicity (the omitted category is non-Hispanic; another included category for which we do not report the estimates is “missing ethnicity information”); and *Poverty*_*i*_ is an indicator equal to one if the mother’s income was at or below 100% of the federal poverty line, and zero otherwise.

These regressions also include fixed effects for age category ( < = 19, 20–24, 25–29, 30–34, 35–39, >=40) because the incidence of PPD varies with age; an indicator is equal to one if the year is 2018 and zero if it is 2017 to control for time effects that affect all zip codes; and zip code fixed effects. The inclusion of zip code fixed effects controls for fixed, unobserved, zip-code specific factors and means that the model identifies the relationship between treatment and changes in screening *within* zip codes. Standard errors are clustered at the level of the zip code to account for possible correlations between omitted variables within zip codes.

These models are estimated both for the overall sample, and for several subsamples. First, women are divided into thirds defined by the 2018 screening rates in their zip code. The purpose of this analysis is to ask whether higher area-level screening rates are associated with greater movement of women into treatment. Second, women are divided by the fraction of the population that identifies as non-Hispanic Black or Hispanic in their zip code in order to see whether higher screening rates have different impacts by race/ethnicity. Third, women are divided by the fraction in poverty in their zip code. The purpose of this analysis is to see whether increases in caregiver screening are more or less strongly associated with treatment in low-income and minority areas.

The main coefficient of interest in these models in *β*_*1*_. In order for *β*_*1*_ to represent a causal effect of zip-code-level increases in PPD screening rates on treatment for PPD, it would have to be the case that there were no omitted variables that were correlated with both zip code PPD screening rates and the probability of individual maternal treatment, conditional on all of the variables included in the model. The inclusion of the mother’s own health history and zip code fixed effects (which control for all of the characteristics of zip codes that remain constant between 2017 and 2018) makes it more likely that this condition holds, but it is ultimately untestable.

Having described the relationship between screening and the treatment of mothers, the next step is to look at whether the screening of an individual mother under her child’s ID was associated with improvements in infant health outcomes. As discussed above, in these analyses the area-level screening rate is used as an instrument for maternal screening in these models, in order to try to estimate a causal effect of screening on child outcomes.

These models take the form:

2 $$\eqalign{& {\rm{Hospitalizatio}}{{\rm{n}}_{{\rm{it}}}}{\rm{ = }}{{\rm{\gamma }}_{\rm{0}}}{\rm{ + }}{{\rm{\gamma }}_{\rm{1}}}{\rm{Pr}}\left( {{\rm{Screene}}{{\rm{d}}_{{\rm{it}}}}} \right){\rm{ + }}{{\rm{\gamma }}_{\rm{2}}}{\rm{Gende}}{{\rm{r}}_{\rm{i}}}{\rm{ + }} \cr & {{\rm{\gamma }}_{\rm{3}}}{\rm{HospBrt}}{{\rm{h}}_{\rm{i}}}{\rm{ + }}{{\rm{\gamma }}_{\rm{4}}}{\rm{LBWorPreter}}{{\rm{m}}_{\rm{i}}}{\rm{ + }}{{\rm{\gamma }}_{\rm{5}}}{\rm{Hispani}}{{\rm{c}}_{\rm{i}}}{\rm{ + }} \cr & {{\rm{\gamma }}_{\rm{6}}}{\rm{InPovert}}{{\rm{y}}_{\rm{i}}}{\rm{ + }}{{\rm{\gamma }}_{\rm{7}}}{\rm{ZipFixedEffect}}{{\rm{s}}_{\rm{z}}}{\rm{ + }}{{\rm{\gamma }}_{\rm{8}}}{\rm{2018 + }}{{\rm{\omega }}_{{\rm{it}}}}{\rm{,}} \cr} $$

where the indicator *Pr(Screened*_*it*_*) *is the predicted probability of the mother being screened under the child’s ID estimated from the following equation:

3 $$\eqalign{& {\rm{Screene}}{{\rm{d}}_{{\rm{it}}}}{\rm{ = }}{{\rm{\delta }}_{\rm{0}}}{\rm{ + }}{{\rm{\delta }}_{\rm{1}}}{\rm{ScreeningRat}}{{\rm{e}}_{{\rm{zt}}}}{\rm{ + }}{{\rm{\delta }}_{\rm{2}}}{\rm{Gende}}{{\rm{r}}_{\rm{i}}}{\rm{ + }} \cr & {{\rm{\delta }}_{\rm{3}}}{\rm{HospBrt}}{{\rm{h}}_{\rm{i}}}{\rm{ + }}{{\rm{\delta }}_{\rm{4}}}{\rm{LBWorPreter}}{{\rm{m}}_{\rm{i}}}{\rm{ + }} \cr & {{\rm{\delta }}_{\rm{5}}}{\rm{Hispani}}{{\rm{c}}_{\rm{i}}}{\rm{ + }}{{\rm{\delta }}_{\rm{6}}}{\rm{InPovert}}{{\rm{y}}_{\rm{i}}}{\rm{ + }} \cr & {{\rm{\delta }}_{\rm{7}}}{\rm{ZipFixedEffect}}{{\rm{s}}_{\rm{z}}}{\rm{ + }}{{\rm{\delta }}_{\rm{8}}}{\rm{2018 + }}{{\rm{v}}_{{\rm{it}}}} \cr} $$

In these models, *Hospitalization*_*it*_ is a measure of whether the child was ever admitted to hospital between 4 and 180 days after birth. *ScreeningRate*_*zt*_ is defined as above, with the exception that the screening rate used as an instrument is computed excluding the child’s own mother (a “leave-one-out” mean). *Gender*_*i*_ is an indicator equal to one if the child is female; *HospBrth*_*i*_ is an indicator equal to one if the child was hospitalized at birth for at least seven days; *InPoverty*_*i*_ is an indicator equal to one if the child’s family is at or below the federal poverty level and zero otherwise; and *LBWorPreterm*_*i*_ is an indicator for whether an infant was born low birthweight (birth weight less than 2500 g) or preterm (less than 37 weeks of gestation). See Table S1 for details about how we identified low birthweight and preterm infants. ZipFixedEffects_z_ is a vector of zip-code-level fixed effects.^4^

The main coefficient of interest in Eq. (2) is *γ*_*1*_ which can be interpreted as the effect of a woman being screened on the probability that her infant is hospitalized. Whether screening has any impact on hospitalization presumably depends on whether screening leads to treatment. If it does not, then one would expect *γ*_*1*_ to be insignificantly different than zero.

In addition, we show estimates of reduced from models that take the form:

4 $$\eqalign{& {\rm{Hospitalizatio}}{{\rm{n}}_{{\rm{it}}}}{\rm{ = }}{{\rm{\alpha }}_{\rm{0}}}{\rm{ + }}{{\rm{\alpha }}_{\rm{1}}}{\rm{ScreeningRat}}{{\rm{e}}_{{\rm{zt}}}}{\rm{ + }} \cr & {{\rm{\alpha }}_{\rm{2}}}{\rm{Gende}}{{\rm{r}}_{\rm{i}}}{\rm{ + }}{{\rm{\alpha }}_{\rm{3}}}{\rm{HospBrt}}{{\rm{h}}_{\rm{i}}}{\rm{ + }} \cr & {{\rm{\alpha }}_{\rm{4}}}{\rm{LBWorPreter}}{{\rm{m}}_{\rm{i}}}{\rm{ + }}{{\rm{\alpha }}_{\rm{5}}}{\rm{Hispani}}{{\rm{c}}_{\rm{i}}}{\rm{ + }} \cr & {{\rm{\alpha }}_{\rm{6}}}{\rm{InPovert}}{{\rm{y}}_{\rm{i}}}{\rm{ + }}{{\rm{\alpha }}_{\rm{7}}}{\rm{ZipFixedEffect}}{{\rm{s}}_{\rm{z}}}{\rm{ + }}{{\rm{\alpha }}_{\rm{8}}}{\rm{2018 + }}{{\rm{\omega}}_{{\rm{it}}}} \cr}. $$

If the IV zip-code level screening rates matter only because they affect an individual mother’s probability of treatment, then the reduced form estimate coefficient *α*_*1*_ should be the IV estimate times the coefficient *δ*_*1*_ from Eq. (3), which measures estimated effect of local screening rates on the probability that an individual woman is screened.

These models are estimated using the full sample. Additionally, mothers may be more likely to have access to treatment (through, for example, alternative non-Medicaid coverage after the first 60 days after giving birth) if they are above the poverty line. Since Michigan Medicaid had stricter income eligibility thresholds for postpartum women than infants, there are few women with incomes above the poverty line in the sample of postpartum women, but there is a sizeable sample of covered infants whose mothers had incomes above the poverty line. Hence, we can also split the child sample by whether mothers had incomes above or below the federal poverty line to ask whether the effects of individual mothers having been screened are different in these two groups.

## Data

The maternal PPD screenings covered by the policy are those that were billed to the child’s Medicaid identification number under the CPT code 96161 (“caregiver health risk assessment”) [12]. Billing under the child’s ID for services provided to the caregiver is allowed under the child’s Early and Periodic Screening, Diagnostic and Treatment (EPSDT) benefit if the care that is provided benefits the child. For mothers who were screened under their own Medicaid identification codes, a different set of CPT codes were used, as shown in Table S1. As shown in Table S2, there was no substantial increase or decrease in the number of women screened for depression under their own Medicaid IDs between 2017 and 2018, while the number of infants whose caregiver was screened under the code 96161 roughly doubled during 2017–2018 (see Table S3).

Some infants in the Medicaid claims data for Michigan and other states share an ID with their mother up to 45 days after birth [24]. According to one CMS report [25], about 18% of IDs associated with maternal delivery in Michigan in 2016 had an associated service that only a newborn could receive. Our analysis includes 96161 screenings billed to infant Medicaid IDs and as well as a few 96161 screenings billed to postpartum women in cases when infants did not yet have their own Medicaid IDs. However, 96.3% of all 96161 screenings in 2018 were billed to infants with unique IDs and 87.8% of all 96161 screenings in 2017 were billed to infants with unique IDs.

The caregiver screening rate at the zip code-year level is constructed as follows: The numerator is the number of unique infants residing in each 5-digit zip code who were born in the first six months of the year and who had at least one caregiver screening billed to their ID in the first six months of life (these procedures are coded 96161 in the claims data). The denominator is the number of postpartum women residing in each zip code who had a delivery in the first six months of the year. Zip code of residence is available for both infants and mothers which allows the screening rate to be computed at the zip code level in this way.

Mother IDs are not linkable to child IDs, so we do not observe whether a particular screening billed to a child’s ID led to a particular mother being treated. That is, we can’t determine whether an individual mother who is treated was screened under the policy or whether someone screened under the policy went on to be treated. However, we can see whether a child whose mother was screened has better outcomes (with the presumed mechanism being via an increased probability that she was treated). The infant level analysis uses a “leave-one-out” zip-code-level screening rate constructed by removing the index child from the calculation.

In order to compare caregivers wholly unaffected by the policy to those who were affected, mothers who gave birth between January 1st, 2017, and June 30th, 2017, are compared to those who delivered between January 1st, 2018, and June 30th, 2018. Excluding women who delivered in 2017 but after June 30th allows “partially exposed” women, who were exposed to the guidelines for only part of their postpartum period, to be excluded. Women who delivered after June 30th 2018 are also excluded so that the analysis compares only mothers who delivered during the first six months of both years. This restriction is potentially important given the possibility of seasonal effects on screening probabilities, treatment, and outcomes. The restriction also allows each caregiver to be followed for at least six months postpartum. Empirically, most of the postpartum screenings observed do fall within this six-month window, as shown in Table S4.

For all caregivers in the sample, information from their Medicaid claims up to 1.5 years prior to delivery is used to control for a history of mental health treatment in the period leading up to the pregnancy and delivery. Specifically, we look for filled prescriptions of antidepressants, antianxiety drugs, antipsychotics, sedatives, or stimulants in up to 1.5 years prior to the delivery. Due to data limitations, it is only possible to examine psychotherapy up to six months prior to delivery. Treatment after delivery is assessed by looking at claims up to six months after delivery to identify new prescriptions for antidepressants or the commencement of psychotherapy.

To examine the effect of the policy on infants, a sample of infants was constructed by including all infants identified in the Medicaid Demographic Eligibility file by birth date. Only infants with a unique Medicaid ID at some point during the birth year are included. Since most infants are assigned their own Medicaid ID in the first 45 days and added to the Demographic eligibility file with this new ID, this approach allows most infants to be included [24].

Zip-year cells with fewer than 11 postpartum women and infants were excluded from the analysis. Although the primary geographic level of analysis – and the smallest geographical unit in our data – is a 5-digit zip code of residence, data from 5-digit zip codes with a population of less than 10,000 were aggregated to the 3-digit zip code level to reduce noise in the estimated rates.

## Results

**Fig. 1:**
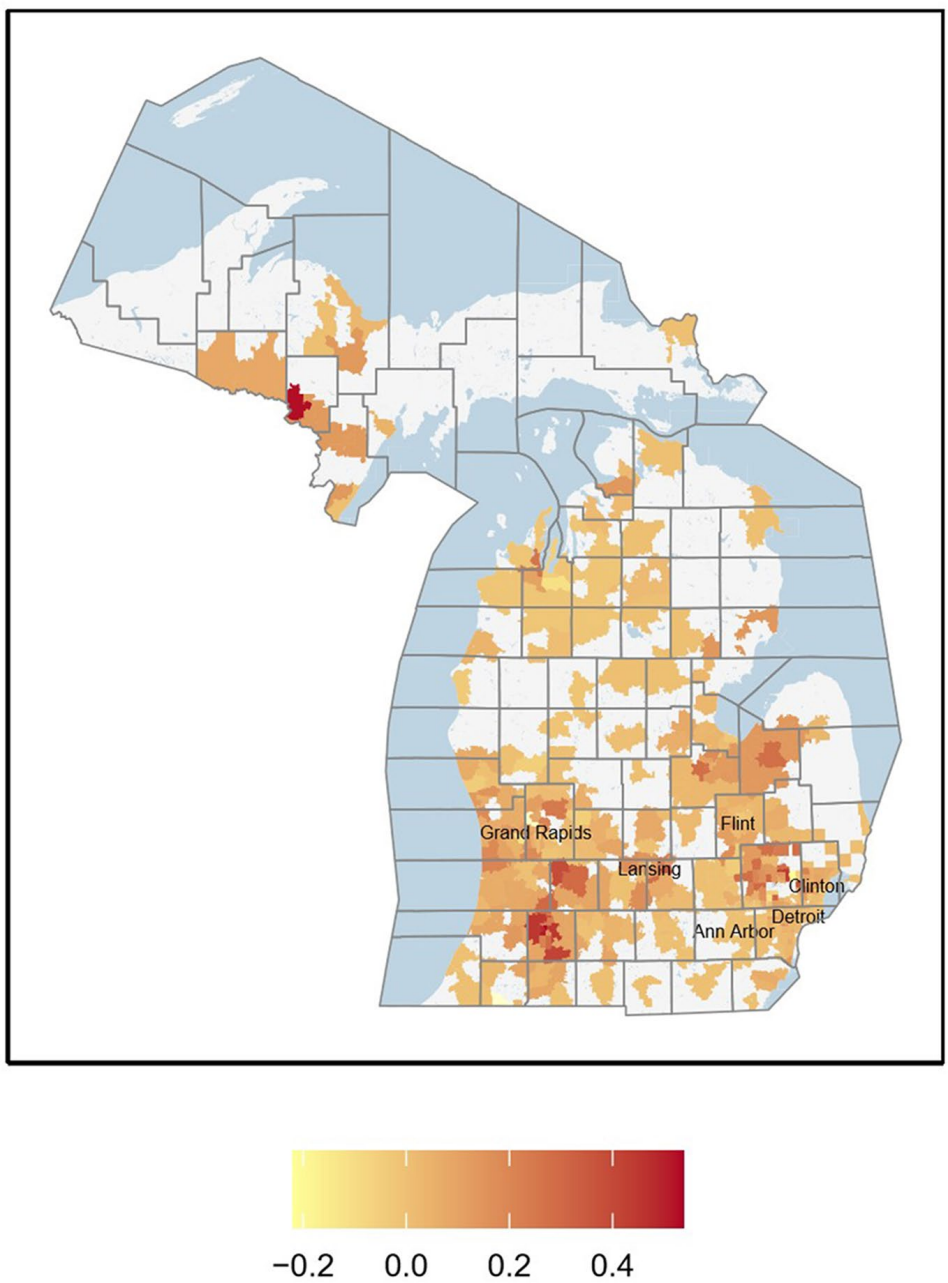
Change in maternal screenings billed to the child’s identification number between 2017 and 2018

Figure 1 shows the change in screenings billed to the child’s Medicaid ID under the new policy between 2017 and 2018. Figure 1 shows that there was an increase in screenings, but it was far from universal and not uniform across geographic areas. Mothers in zip codes with the highest screening rates under the new program were disproportionately located in areas with relatively low population densities, low percentage in poverty, and low percentages of Black population as shown in Table S5 which presents the mean characteristics of zip codes in the top, middle, and bottom thirds of screening rates under the child’s ID in 2018. The mean screening rate in these high screening zip codes was 13.9% across both years and 20.1% in 2018 (where a person is counted as screened if even one screening at a child’s well visit took place) suggesting far from complete compliance with screening recommendations even after the new billing policy (Figure S1 in the Additional File shows zip-code-level variation in the screening rate in 2018).

Table 1 shows sample means for mothers and children in 2017 and 2018. The numbers for mothers (Panel A) suggest that there were only small changes in the characteristics of mothers between 2017 and 2018: In 2018 mothers in these areas were slightly more likely to have a depression history, full-year Medicaid coverage, and were somewhat older.

**Table 1:** Mean characteristics of individual mothers and children, 2017 and 2018

	All zip codes	
**A. Mothers**	**Year = 2017**	**Year = 2018**
Any depression treatment	0.155	0.156
Depression history	0.114	0.124
Other mental health history	0.060	0.063
Full year Medicaid coverage	0.742	0.747
Income below poverty	0.882	0.882
Hispanic	8.68	8.52
Ethnicity unknown	6.31	6.39
Age < = 19	5.89	5.67
Age 20–24	29.45	28.28
Age 25–29	34.07	34.26
Age 30–34	19.62	20.36
Age 35–39	8.55	8.88
Age > = 40	2.42	2.55
Number of mothers	23,112	22,393
**B. All children**	**Year = 2017**	**Year = 2018**
Hospitalized 4-180 days after birth	0.049	0.047
Caregiver screened in 1st 6 months*	0.025	0.062
Hospital stay at birth > = 7 days	0.062	0.059
Birthweight < 2500 g or gestation < 37 weeks	0.088	0.086
Female	0.488	0.488
Hispanic	7.90	7.47
Missing ethnicity	12.10	13.94
At/Below poverty	0.709	0.707
Number of children	32,259	31,671
**C. Children with mother screened**	**Year = 2017**	**Year = 2018**
Hospitalized 4-180 days after birth	0.059	0.049
Hospital stay at birth > = 7 days	0.078	0.071
Birthweight < 2500 g or gestation < 37 weeks	0.117	0.087
Female	0.506	0.494
Hispanic	15.06	10.29
Missing ethnicity	8.53	10.90
At/Below poverty	0.681	0.685
Number of children	797	1,972

An asterisk indicates that the estimate is statistically significant at the 95% level of confidence. A double asterisk indicates that it is significant at the 99% level of confidence. The fact that fewer children than mothers are in households with incomes less than the poverty line indicates that Medicaid coverage of children is more generous than coverage of mothers in Michigan

Panel B confirms that there was a noticeable increase in screening rates: In 2017, 2.5% had a caregiver screened compared to 6.2% in 2018. Otherwise, child characteristics remained very similar.

Panel C focuses on children whose mother was screened at least once in the first six months after giving birth. Compared to children born in 2017, screened children born in 2018 are less likely to be born low birthweight or preterm or to be of Hispanic ethnicity, suggesting that the scope of screenings broadened.

Estimates of Eq. (1), which shows the relationship between maternal PPD treatment and zip-code PPD screening rates under the new policy, are shown in Table 2 for all mothers and for those in each tercile of the 2018 screening rate distribution.

**Table 2:** Probability that mothers received depression treatment as a function of the screening rate in the zip code of residence

Sample→ Variable ↓	(1) All Mothers	(2) 2018 screening rate tercile 3	(3) 2018 screening rate tercile 2	(4) 2018 screening rate tercile 1
Zip code level screening rate	0.016 (0.033)	0.088* (0.040)	-0.044 (0.238)	0.191 (0.280)
Any depression history	0.408** (0.009)	0.409** (0.014)	0.414** (0.015)	0.403** (0.016)
Other mental health history	0.110** (0.010)	0.118** (0.016)	0.109** (0.017)	0.100** (0.018)
Full year Medicaid	0.054** (0.004)	0.053** (0.006)	0.063** (0.007)	0.047** (0.006)
Hispanic	-0.043** (0.007)	-0.046** (0.009)	-0.043** (0.016)	-0.037** (0.013)
At/Below poverty	0.024** (0.004)	0.028** (0.007)	0.030** (0.008)	0.014 (0.008)
Age group FEs included	yes	yes	yes	yes
Zip fixed effects included	yes	yes	yes	yes
Control for year = 2018	yes	yes	yes	yes
Number observations	45,505	15,044	15,094	15,367
Depression screening rate, 2017	0.034	0.077	0.021	0.005
Depression screening rate, 2018	0.088	0.201	0.054	0.010
Maternal treatment rate, 2017	0.155	0.175	0.143	0.146
Maternal treatment rate, 2018	0.156	0.169	0.153	0.146

The table shows estimates of Eq. (1). The sample includes all postpartum women with Medicaid coverage for 2017 and 2018, who lived in zip-year cells with at least 11 observations. Treatment is defined as having a claim for a new filled antidepressant prescription (not a refill) or a claim for psychotherapy (including family therapy – see Table S1 for detail) or both in the first six months after delivery. Robust standard errors clustered at the zip code level are shown in parentheses. An asterisk indicates that the estimate is statistically significant at the 95% level of confidence. A double asterisk indicates that it is significant at the 99% level of confidence.

The first column of Table 2 shows that increases in the area-level screening rate had no statistically significant impact on the probability that a mother was treated for depression postpartum. The other variables have the expected signs: A recent depression history is strongly predictive of postpartum depression treatment, while a recent history of other mental health disorders has a smaller effect. Mothers with full year Medicaid coverage are more likely to receive treatment, as are women with incomes at or below poverty, which may indicate higher rates of PPD in these groups. Finally, Hispanic ethnicity is associated with lower treatment rates as has been previously reported [3].

Given that screening was not universal, a possible explanation for these patterns is that only mothers with the most obvious symptoms of PPD were likely to be screened during well child visits, and these women were likely to receive treatment in any case. However, places with higher screening rates may also be more likely to detect cases that would not have otherwise been detected.

To test this hypothesis, columns 2 to 4 of Table 2 divide mothers into terciles by area-level caregiver screening rates in 2018. The estimates show that for mothers in areas in the top tercile of screening during well-child visits, there is a positive relationship between screening rates and the probability of treatment. Given the screening rate of 20.1% in these zip codes in 2018, the coefficient estimate of 0.088 translates into a 10.1% increase in treatment (0.088 times 0.201 divided by 0.175).

Table S5 showed that areas with high concentrations of individuals in poverty and of minority women were less likely to experience increases in screening under the new rule. However, given higher underlying rates of PPD in these groups and greater possible difficulties accessing depression treatment, increases in screening might have different effects in high minority and high poverty areas. This question can be addressed by dividing women by the fraction of the zip code that is minority and the fraction of the zip code that is in poverty. These estimates are shown in Table 3.

The first three columns of Table 3 show that zip codes in the top third by share minority had a minority share of 70.1% in the full sample, compared to 19.3% in the next highest third, and 4.88% in the lowest minority tercile. Although the screening rate in high minority areas was low, increases in screening associated with the policy change significantly increased the probability that mothers received PPD treatment. The coefficient estimate suggests that the increases in screening in the highest minority zip codes increased the probability of treatment by 7.3% (0.101 times 0.077 divided by 0.106). Since rates of PPD are thought to be higher among minority women, this estimate suggests that these women may have difficulties accessing treatment after screening positive for PPD. Similarly, the last three columns of Table 3 suggest that mothers in the zip codes with the highest percent of individuals in poverty also experience increases in PPD treatment when screening rates increase. The point estimate suggests that the increase in screening increased treatment rates by 8.3% (0.115 times 0.071 divided by 0.098).

**Table 3:** Probability that mothers received depression treatment as a function of zip code screening rates by the percent minority in the zip code of residence and the percent in poverty in the zip code

Sample→ Variable ↓	(1) Highest % minority		(2) Middle % minority	(3) Lowest % minority	(4) Highest % in poverty	(5) Middle % in poverty	(6) Lowest % in poverty
Zip code level screening rate	0.101* (0.040)		0.043 (0.037)	-0.122 (0.081)	0.115** (0.033)	-0.027 (0.068)	-0.013 (0.056)
Any depression history	0.384** (0.020)		0.416** (0.015)	0.415** (0.013)	0.382** (0.018)	0.391** (0.016)	0.441** (0.012)
Other mental health history	0.092** (0.016)		0.109** (0.016)	0.125** (0.018)	0.096** (0.014)	0.111** (0.016)	0.123** (0.019)
Full year Medicaid	0.043** (0.006)		0.048** (0.005)	0.072** (0.007)	0.038** (0.005)	0.059** (0.007)	0.064** (0.005)
Hispanic	-0.028** (0.009)		-0.056** (0.011)	-0.045** (0.013)	-0.034** (0.010)	-0.049** (0.013)	-0.046** (0.010)
Below poverty	0.025** (0.006)		0.020* (0.008)	0.028** (0.008)	0.021** (0.005)	0.036** (0.008)	0.016* (0.008)
Age group FEs included	yes		yes	yes	yes	yes	yes
Zip fixed effects included	yes		yes	yes	yes	yes	yes
Control for year = 2018	yes		yes	yes	yes	yes	yes
Number of observations	14,955		15,332	15,218	14,924	14,984	15,597
Screening rate, 2017	0.039		0.032	0.031	0.035	0.027	0.039
Screening rate, 2018	0.077		0.101	0.086	0.071	0.078	0.113
Maternal treatment rate, 2017	0.106		0.153	0.204	0.098	0.185	0.180
Maternal treatment rate, 2018	0.101		0.164	0.201	0.095	0.187	0.183
Mean % minority in zip code	70.1		19.3	4.88			
Mean % in poverty in zip code					35.5	18.5	10.3

This table reports estimates from Eq. (1). The sample includes all postpartum women with Medicaid coverage for 2017 and 2018, from zip-year cells with at least 11 observations. Treatment is defined as having a claim for a new filled antidepressant prescription (not a refill) or a claim for psychotherapy (including family therapy – see Table S1 for detail) or both in the first six months after delivery. Percent minority is defined as percent non-Hispanic Black plus percent Hispanic at the zip code level. Robust standard errors clustered at the zip code level are shown in parentheses. An asterisk indicates that the estimate is statistically significant at the 95% level of confidence. A double asterisk indicates that it is significant at the 99% level of confidence.

The main rationale for screening mothers during well child visits is that treating mothers for PPD is also likely to benefit the child. This hypothesis can be tested by investigating whether screening the mother during a well child visit improves the child’s health outcomes. Models of the relationships between children’s hospitalizations during the first six months after birth and maternal screening in the first six months after delivery are estimated using Eqs. (2)-(4) and the results appear in Table 4.

The first two columns show estimates for the full sample of infants. Reduced form estimates are shown in the odd-numbered columns and the IV estimates are shown in the even-numbered columns in Table 4. The coefficient of interest is the coefficient on the “leave-one-out” screening rate for the reduced form models and the coefficient on the indicator for whether the child’s own mother screened for the IV models. The IV estimate for the full sample of children suggests that screening mothers for PPD at well child visits reduced their infant’s probability of hospitalization but neither the IV estimate nor the reduced form showing the impact of area-level screening rates more generally are statistically significant.^5^

Next, we split the sample by the poverty status of the child’s family. The mean characteristics of infants in these two samples (above vs. below 100% of the federal poverty line) are shown in Table S6. There are roughly three times more infants above the poverty line in our sample than mothers (5,370, or 11.8% of postpartum women vs. 18,679, or 29.2% of infants above the poverty line), because Michigan Medicaid is more generous for infants than for pregnant women. In other words, our sample contains a relatively large number of infants (the majority of whose mothers we do not observe in our data) who are above the poverty line and therefore whose mothers likely have better access to treatment than the mothers of infants below the poverty line (all of whom we observe in our data). These estimates are shown in columns 3–6.

The estimates show that maternal screening is associated with statistically significant reductions in infant hospitalizations only in the sample of infants who are above 100% of the federal poverty line. We hypothesize that the mothers of these infants may have been more likely to have access to treatment if they screened positive. In this sample, the IV estimates suggest that observed increases in the probability of screening among infants who were not in poverty reduced the probability of hospitalization by 18.1%. The reduced form estimates indicate that the observed increase in zip-code-level screening rates among these infants reduced hospitalizations by 7.7%.^6^ Given the estimates of Eq. (3) shown in Table S7, these estimates are consistent with the idea that increases in zip-code-level screening operated mainly through increasing the probability of maternal treatment.

**Table 4:** Effects of caregiver screenings on infant hospitalizations in the first six months

Sample→ Variable ↓	All		In Poverty		Not in Poverty		
	(1) Reduced form	(2) IV	(3) Reduced form	(4) IV	(5) Reduced form	(6) IV	
Leave-one-out screening rate	-0.013 (0.014)		-0.007 (0.020)		-0.028* (0.012)		
Child’s own mother was screened		-0.030 (0.030)		-0.015 (0.043)		-0.066* (0.031)	
Stayed in hospital at birth for at least 7 days	0.067** (0.006)	0.068** (0.006)	0.075** (0.007)	0.075** (0.007)	0.036** (0.012)	0.036** (0.012)	
Birthweight < 2500 g or gestation < 37 weeks	0.041** (0.006)	0.041** (0.006)	0.040** (0.007)	0.040** (0.007)	0.043** (0.011)	0.044** (0.011)	
Female	-0.010** (0.001)	-0.010** (0.001)	-0.011** (0.002)	-0.011** (0.002)	-0.007* (0.003)	-0.007* (0.003)	
Hispanic FE	-0.009** (0.003)	-0.008** (0.003)	-0.012** (0.004)	-0.012** (0.004)	-0.000 (0.005)	0.001 (0.005)	
At/Below poverty	0.012** (0.002)	0.012** (0.002)					
Zip code fixed effects	yes	yes	yes	yes	yes	yes	
Control for year = 2018	yes	yes	yes	yes	yes	yes	
Number observations	63,930		45,251		18,679		
Hospital. rate, 2017	0.049		0.055		0.035		
Hospital. rate, 2018	0.047		0.051		0.037		
Screening rate, 2017	0.035		0.034		0.037		
Screening rate, 2018	0.090		0.088		0.096		
F-statistic for IV		118.5		72.1		115.9	

The reduced form models take the form of Eq. (4). The IV models are based on Eqs. (2) and (3). The first stage estimates from the IV models are shown in the Additional File Table S7. The dependent variable is equal to one if there is any infant hospitalization record in the Inpatient file with an admission date 4-180 days after birth (i.e. not including the initial hospitalization at birth, if any). Robust standard errors clustered at the zip code level are shown in parentheses. An asterisk indicates that the estimate is statistically significant at the 95% level of confidence. A double asterisk indicates that it is significant at the 99% level of confidence.

## Discussion

This study focuses on three questions: Whether allowing pediatric providers to bill Medicaid for PPD screenings during well child visits increased compliance with recommendations to screen mother during these visits; whether screening mothers during well child visits was associated with an increase in treatment for PPD; and whether infants whose mothers were screened during well child visits had fewer hospitalizations.

We find that the policy was associated with increases in screening rates, although take up was very uneven and overall screening rates remained low. This finding suggests that additional measures to increase compliance with screening recommendations might help to better integrate maternal screening into pediatric clinical practice.

Findings about the effects of screening on treatment were also mixed, with little overall increase in treatment rates. However, in areas that were in the top third in terms of area screening rates, higher screening was associated with higher maternal treatment rates. One interpretation of this result is that in areas where overall screening rates remained low, only the most severe cases of PPD were detected and these cases might have been likely to be treated in any case. This interpretation suggests that screening rates must surpass some critical threshold before the screens start detecting significant numbers of cases that would not have been detected and treated otherwise. Although screening rates in areas with high fractions of women in poverty and/or minority women were low, screening was more likely to yield increases in treatment in these areas. This observation suggests a missed opportunity to better serve the most vulnerable populations of new mothers.

Turning to infant health, we find that among infants of women who were not in poverty, the observed increases in screening decreased hospitalizations by 7.7% in the first six months of life (not including hospitalizations at birth, if any). We hypothesize that the main mechanism for this is that women who are screened are more likely to be treated when they are not in poverty. These results may suggest that there are barriers in accessing PPD treatment and that screening for PPD at well child visits has the potential to reduce infant hospitalizations only if the effort is accompanied by expansion of access to treatment.

This study has several limitations, largely due to limitations of the available data. First, some PPD screenings might occur *during a postpartum visit* and not be billed as a separate service. However, screenings during postpartum visits were not the focus of the policy, and there is little reason to think that such screenings increased substantially between 2017 and 2018 or the way such visits were billed changed systematically between 2017 and 2018. Although we cannot directly evaluate the number of such screenings that were *not* billed as a separate service, we show in Table S2 that there was little change in the screening rate for depression in postpartum women in 2017–2018 when the screening *was* billed as a separate service.

Second, the code that identifies caregiver screening during well child visits (Current Procedural Terminology (CPT) code 96161) could be used to bill for other types of caregiver health risk assessment screenings. This is likely to be the reason that some zip codes already had small numbers of providers using this code in 2017 when it was introduced. However, it is reasonable to assume that most of the increase in use of this code was driven by increases in PPD screening due to the new billing policy. Our findings about the relationship between caregiver screening and treatment for PPD support this view, though we cannot attribute 100% of screenings billed to the child’s Medicaid ID in 2018 to PPD screenings. The only other screening recommendation involving infants that was made at the same time was a recommendation to screen infants for hyperbilirubinemia. This condition is common in infants and is usually harmless, so it is unlikely to have driven changes in infant hospitalization rates.

A third limitation is that mother and child IDs could not be linked. Hence, it is not possible to know whether screening a specific mother during a well child visit led her to get treatment (because such screenings are billed to the child ID and not the mother ID). Importantly, we can still see whether a mother’s screening that was billed under her child’s ID is related to the child’s health.

A fourth limitation is that roughly 20% of Michigan infants share a Medicaid ID with their mother for up to 45 days in the first year of life, though there may be cases where a newborn stays on their mother’s Medicaid ID for longer [24, 25]. Hospitalizations may be undercounted for these infants in the first 45 days of life because they would be billed to the mother’s ID. However, we are not aware of any reason why an infant sharing an ID with their mother would have been affected by the change in the screening billing rule.

A fifth limitation is that information about race and ethnicity is missing for most claims, so that we focus on the racial and ethnic composition of neighborhoods rather than individuals.

A sixth limitation is the way in which we define PPD treatment and mental health history. First, we exclude depression medication refills from our definition of PPD treatment to avoid overestimating PPD treatment. Since a refill could represent continuation of pre-pregnancy depression treatment, we do not want to incorrectly count it as PPD treatment. We do likely miss some PPD treatment this way, but we think that is preferrable to overstating the extent of PPD treatment. Second, we do not observe pre-pregnancy mental health treatment information for the same length of time for all women.

This study has examined the relationship between a rule allowing pediatric providers to bill Medicaid for PPD screenings provided during well-child visits and screening rates, PPD treatment, and infant hospitalizations. A strength of the analysis is that it includes all postpartum mothers affected by the rule and their children. The estimates show that the impact of the rule varied considerably from place to place and for different groups of women. Hence, analyses based on small samples from specific locations or groups could come to widely different conclusions about the effects of the state-wide rule. This variation in the take up of the rule is deserving of further research.

## Supplementary Information

Below is the link to the electronic supplementary material.

Supplementary Material 1

Supplementary Material 2

## Data Availability

The TAF Research Identifiable Files (TAF-RIF) are created by CMS and available by application to qualified researchers. The code used to create our analytic extracts and perform our analysis will be made publicly available in a dataverse repository.

## Footnotes

1 Although this policy has been referred to as a requirement [16], we refer to it as a recommendation because, to the best of our knowledge, there is no monitoring of providers or enforcement of compliance.

2 Although the literature on the consequences of maternal postpartum depression sometimes considers additional outcomes such as, for example, the timely completion of vaccinations [9], we cannot examine such longer-term outcomes due to data limitations.

3 For example, the income cutoff for pregnant women is 200% of the federal poverty line compared to 217% for infants. Other differences are that special needs children are covered at even higher income levels and recent immigrants were not covered for pregnancy, while their citizen infants were covered.

4 We include 3-digit fixed effects for all zip codes and additional fixed effects for each 5-digit zip code with a population of at least 25,000.

5 0.013 (0.030) times the 2018 screening rate of 0.090 divided by the baseline hospitalization rate of 0.049.

6 The reduced form estimate of 0.028 divided by the baseline hospitalization rate of 0.035, multiplied by 0.10.
