# Supplementary material for: Screening for postpartum depression at well child visits: evaluating the impact of Michigan’s statewide initiative
Source: Health Econ Rev. 2025 Aug 26;15:72. doi: 10.1186/s13561-025-00671-2 (PMC12379358; doi:10.1186/s13561-025-00671-2)
Supplement: Supplementary file 1 — Supplementary Material 1 [file 13561_2025_671_MOESM1_ESM.docx]

Additional File 1 for

**Screening for Postpartum Depression at Well Child Visits: Evaluating the Impact of Michigan’s Statewide Initiative**

Janet Currie

Anna Malinovskaya

**This file includes:**

Supplementary Text

Figure S1

Tables S1 to S7

References (1 to 3)

**Supplementary Text**

**Details regarding the identification of postpartum mothers**

To identify postpartum women in the Medicaid claims files, we followed the approach described as “Approach 4” in Auty et al. (1), with minor modifications.

Specifically, first, we selected women of age 14-50 in the Demographic eligibility file for the respective calendar year.

Next, we linked this sample of women with their claims (if any) in the Inpatient File for the same calendar year, and further limited the sample to women with a claim containing any of the following ICD-10 diagnosis codes: O80, O82, Z370, Z372, Z373, Z3750, Z3751, Z3752, Z3753, Z3754, Z3759, Z3760, Z3761, Z3762, Z3763, Z3764, Z3769, Z379, and Z390. These are all maternal delivery diagnosis codes. For postpartum women identified in this way, we defined their delivery date as their hospital admission date on the respective claim.

Next, we linked the sample of women from step 1 above with their claims in the Other Services File for the same calendar year. Although claims from inpatient facilities are usually stored in the Inpatient File, a small fraction of them is stored in the Other Services File. We selected women with claims in the Other Services File for which the place of service is listed as a hospital and which contain diagnosis code O80 or O82 (limiting the set of diagnosis codes to these two for maternal delivery claims found in the Other Services File gave us the closest match between the number of postpartum women identified in each calendar year and the publicly reported number of live births financed by Medicaid in the respective calendar years). For these women, the date of delivery was defined as the date the associated procedure on the claim was performed. If a woman was linked with multiple claims with relevant diagnosis codes but different dates, we selected the claim with the earliest date.

Finally, we combined the two sub-samples of postpartum women obtained above. If a woman was linked with relevant claims in both the Inpatient File and the Other Services File, we kept her record from the Inpatient File only.

Although our approach follows Auty et al.’s approach 4, there are a few minor differences (1). First, Auty et al. employ revenue center codes in their approach, whereas we do not use them (1). Second, while Auty et al. use the full set of diagnosis codes listed above to identify postpartum women in the Other Services File, we use only two diagnosis codes to identify postpartum women in the Other Services File. The number of postpartum women we identified matches closely with the publicly reported number of women on Medicaid who gave birth in Michigan in those years.

**Other details**

The data for this study come from two sources. The first is the TAF Research Identifiable Files (TAF-RIF) for Michigan for 2017-2018. Files from 2016 are also used to measure mental health histories, as discussed further below. The TAF-RIF are files derived from Medicaid health insurance claims data which CMS makes available to qualified researchers. The file structure is complex involving a file with demographic characteristics (the Demographic Eligibility file), the Inpatient file, the Other Services file, and the Rx file, which can be linked together with an anonymized patient identifier. Although the data does not contain personal identifiers, because the TAF-RIF are derived from underlying confidential health claims, our study protocol was vetted by Princeton University’s Institutional Review Board.

The second source of data is publicly available information about annual zip-code-level measures of social determinants of health from the Agency for Healthcare Research and Quality’s Social Determinants of Health database (2). This data is used to investigate which zip code level characteristics are associated with higher compliance with the policy (i.e., larger increases in screenings of caregivers during pediatric visits between 2017 and 2018).

Drugs were classified into Anatomical Therapeutic Chemical types (e.g. antidepressants) according to their National Drug Codes using the RxNorm/RxMix databases made available by the National Institutes of Health.

A significant limitation of the data is that the information about mother and infant race is overwhelmingly missing. Fewer than 10% of observations list race. This is why we focus on the percentage minority in the zip code of residence as a descriptor.

Although some 96161 screenings were billed to the infant Medicaid ID in 2017, we believe that only a fraction of them were for postpartum depression screening. The billing code 96161 is not specific to postpartum depression screening (3). The policy in question encouraged providers to use code 96161 for postpartum depression screening during well child visits. Although other types of caregiver screenings billed to the child ID could also increase during 2017-2018, we believe that most of the increases in 96161 screenings billed to the child ID in 2018 were for postpartum depression and are attributable to the policy. To confirm this, we analyze the association between the zip-code level screening rate and PPD treatment among postpartum women.

**Figure S2**: Maternal screenings billed to the child’s identification number in 2018.


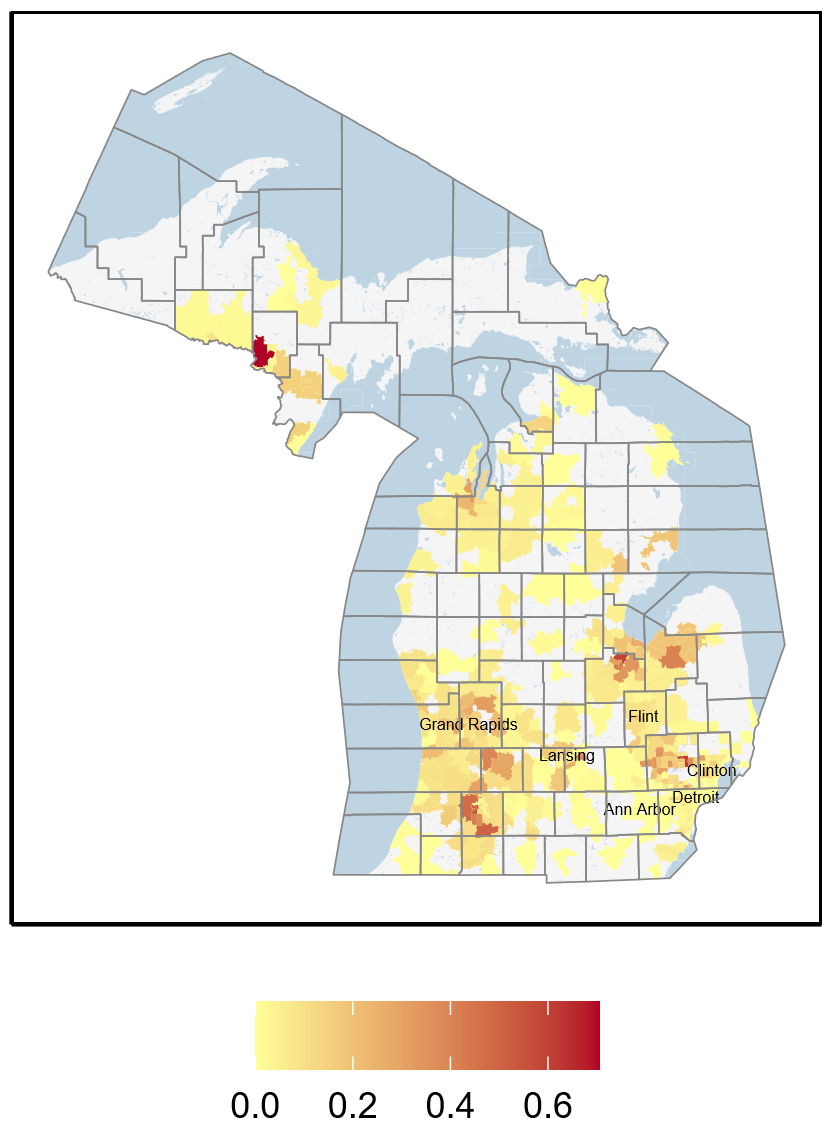


Figure S2 notes: Data is at the 5-digit zip code level. Unshaded areas are those with less than 11 postpartum women after aggregating data from small population zip codes to the 3-digit zip code level.

**Table S1**. CPT / HCPCS codes used to identify adult depression screening and psychotherapy, as well as infants hospitalized at birth for low birthweight and/or preterm birth

| Depression screening codes* | G8431, G8510, G8432, G8433, G8511, G8940, G9717, 99420, 96146, 96127, 96130, 96131, 96132, 96133, 96136, 96137, 96138, 96139, 99394, 99384, 99385, 99395, 99386, 99396, 96101, 96102, 96103, H0031, S3005, G0444. |
| --- | --- |
| Psychotherapy codes | 90832, 90833, 90834, 90836, 90837, 90838, 90839, 90840, 90845, 90846, 90847, 90853, G0017, G0018. |
| Low birthweight / preterm codes | P0700, P0701, P0702, P0703, P0704, P0705, P0706, P0707, P0708, P0709, P0710, P0711, P0712, P0713, P0714, P0715, P0716, P0717, P0718, P0720, P0721, P0722, P0723, P0724, P0725, P0726, P0727, P0728, P0729, P0730, P0731, P0732, P0733, P0734, P0735, P0736, P0737, P0738, P0739. |

Note: *These codes are usually billed directly to the Medicaid ID of the patient being screened in contrast with the CPT code 96161 recommended for use for maternal depression screening at well child visits and usually billed to the child’s Medicaid ID.

**Table S2.** Depression screenings in postpartum women in Michigan billed to women’s own Medicaid IDs

|  | 2017 | 2018 |
| --- | --- | --- |
| Women with a delivery in the first half of the year | 23,436 | 22,755 |
| Of those, women with at least one depression screening in the first 6 months after delivery | 3549 (15.1%) | 3,961 (17.4%) |
| Total number of depression screenings billed to the mother’s Medicaid ID | 4,981 | 5,670 |

**Table S3**. Caregiver health risk assessment screenings under the CPT code 96161 billed to infant Medicaid IDs in Michigan, 2016-2018

| Year of data | 2016 | 2017 | 2018 |
| --- | --- | --- | --- |
| Sample | Infants born in 2015 or 2016 | Infants born in 2016 or 2017 | Infants born in 2017 or 2018 |
| Total caregiver screenings (CPT code 96161) billed to the infant Medicaid ID in the first 6 months | 0 (the code was not yet in use) | 3,171 | 8,104 |
| Unique infants with a caregiver screened (CPT code 96161) in the first 6 months | 0 (the code was not yet in use) | 1,848 | 4,522 |

**Table S4**. Timing of caregiver screenings in Michigan billed to the child’s ID, by year

| Conducted… | 2017 | 2018 |
| --- | --- | --- |
| <=30 days after birth | 1,035 (860 UI) | 2,532 (2,025 UI) |
| >30 and <=60 | 588 (558 UI) | 1,494 (1,405 UI) |
| >60 and <=90 | 894 (852 UI) | 1,990 (1,918 UI) |
| >90 and <=120 | 63 (60 UI) | 238 (229 UI) |
| >120 and <=180 | 590 (538 UI) | 1,848 (1,762 UI) |
| >180 | 683 (592 UI) | 1,907 (1,674 UI) |
| Total unique screening instances | 3,853 | 10,009 |

Note: The universe is all caregiver screenings in Michigan (CPT code 96161) billed to infant Medicaid IDs in 2017 and 2018, respectively. Only screenings billed to infants with a unique Medicaid ID in the Demographic eligibility file for the respective calendar year are included. An infant is defined in this table as anyone who is under 1 year old in the respective calendar year (but could be born in the previous calendar year). These are instances of screenings, not unique infants screened. UI is unique infants *in that bin*, so each infant might appear in more than one bin.

**Table S5**. Means of zip code characteristics for the full sample of mothers in top, middle, and lowest tercile by screening rates in Michigan between 2017 and 2018

|  | Top screening tercile | Middle screening tercile | Lowest screening tercile |
| --- | --- | --- | --- |
| Mean zip code screening rate in 2017-2018, % | 13.9 | 3.68 | 0.73 |
| % In poverty | 18.5 | 21.6 | 23.6 |
| % Non-Hispanic Black | 18.0 | 23.9 | 32.2 |
| % Hispanic | 8.42 | 5.96 | 4.85 |
| % Foreign born | 7.68 | 7.80 | 7.37 |
| Dist. to nearest obstetrics unit | 3.63 | 4.95 | 3.91 |
| Population per sq. mile | 2031 | 2685 | 2791 |
| % Under 65 with Medicaid/means tested public health insurance coverage | 25.3 | 29.8 | 31.2 |
| Number of mothers in the sample | 15,044 | 15,094 | 15,367 |

Note: Zip code characteristics for mother’s zip code of residence come from the AHRQ (2).

Distance is measured in miles.

**Table S6**. The mean characteristics of infants in the sample by poverty status

| Sample→  Variable↓ | Poor | Non-poor | Is the difference statistically significant? |
| --- | --- | --- | --- |
| Caregiver screened in 1^st^ 6 months of life, % | 4.18 | 4.69 | ** |
| Any hospitalization 4-180 days after birth, % | 5.28 | 3.58 | ** |
| Hospitalized for at least 7 days at birth, % | 6.87 | 3.98 | ** |
| Born low birthweight or preterm, % | 9.69 | 6.40 | ** |
| Female, % | 48.8 | 48.7 | No |
| Hispanic, % | 7.66 | 7.77 | No |
| Missing ethnicity information, % | 12.5 | 14.2 | ** |
| Number of observations | 45,251 | 18,679 | - |

Note: Single asterisk indicates significance at the 95% level of confidence. Double asterisk indicates significance at the 99% level of confidence. We classify an infant as poor if their state-defined family income is from 0 to 100% of the federal poverty level (FPL).

**Table S7**. First stage estimates from the IV models in Table 4

| Sample→    Variable ↓ | All | In  poverty | Not in poverty | | |
| --- | --- | --- | --- | --- | --- |
| Leave-one-out screening rate | 0.455**  (0.042) | 0.474**  (0.056) | | 0.421**  (0.039) |  |
| Stayed in hospital at birth for at least 7 days | 0.007  (0.004) | 0.006  (0.004) | | 0.012  (0.011) |  |
| Birthweight<2500g or gestation<37 weeks | 0.002  (0.003) | 0.000  (0.004) | | 0.006  (0.007) |  |
| Female | 0.001  (0.001) | 0.002  (0.002) | | -0.001  (0.003) |  |
| Hispanic FE | 0.014*  (0.006) | 0.016*  (0.007) | | 0.010  (0.008) |  |
| At/Below poverty | -0.003  (0.002) |  | |  |  |
| Zip code fixed effects | yes | yes | | yes |  |
| Control for year=2018 | yes | yes | | yes |  |
|  |  |  | |  |  |
| Number observations | 63,930 | 45,251 | 18,679 | | |

**References**

1. S.G. Auty, J.R. Daw, L.K. Admon, S.H. Gordon, Comparing Approaches to Identify Live Births Using the Transformed Medicaid Statistical Information System. *Health Services Res*earch *59*(1):e14233 (2024).
2. Agency for Healthcare Research and Quality [AHRQ], Social Determinants of Health Database. Rockville, MD (2023). <https://www.ahrq.gov/sdoh/data-analytics/sdoh-data.html>
3. American Medical Association [AMA], Behavioral Health Coding Resource, (2025). <https://www.ama-assn.org/system/files/behavioral-health-coding-resource.pdf>
