# Supplementary material for: Screening for postpartum depression at well child visits: evaluating the impact of Michigan’s statewide initiative
Source: Health Econ Rev. 2025 Aug 26;15:72. doi: 10.1186/s13561-025-00671-2 (PMC12379358; doi:10.1186/s13561-025-00671-2)
Supplement: Supplementary file 2 — Supplementary Material 2 [file 13561_2025_671_MOESM2_ESM.docx]

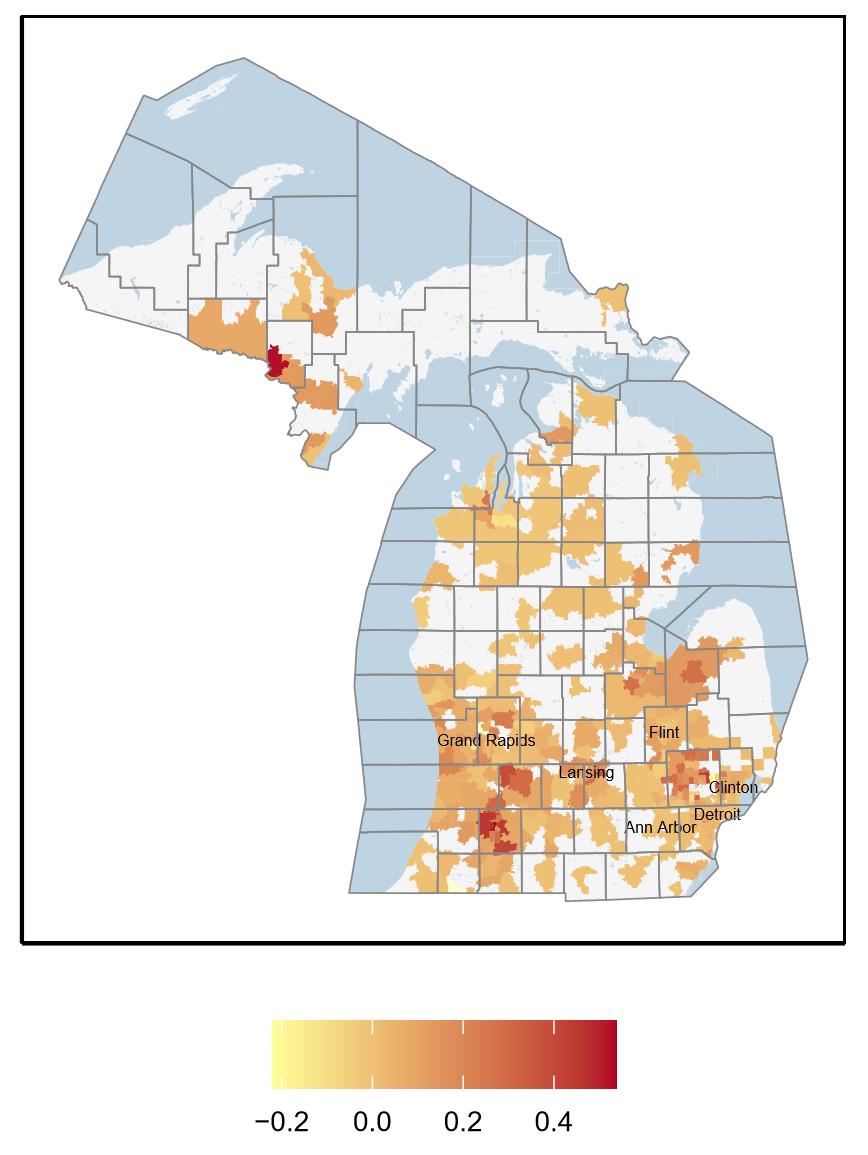


Figure S1 notes: Data is at the 5-digit zip code level. Unshaded areas are those with less than 11 postpartum women after aggregating data from small population zips codes to the 3-digit zip code level.
